# Supplementary material for: spVC for the detection and interpretation of spatial gene expression variation
Source: Genome Biol. 2024 Apr 19;25:103. doi: 10.1186/s13059-024-03245-3 (PMC11027374; doi:10.1186/s13059-024-03245-3)
Supplement: Supplementary file 1 — Additional file 1. Supplementary Figures S1-S24 and Tables S1-S4. [file 13059_2024_3245_MOESM1_ESM.pdf]

Supplementary Figures and Tables

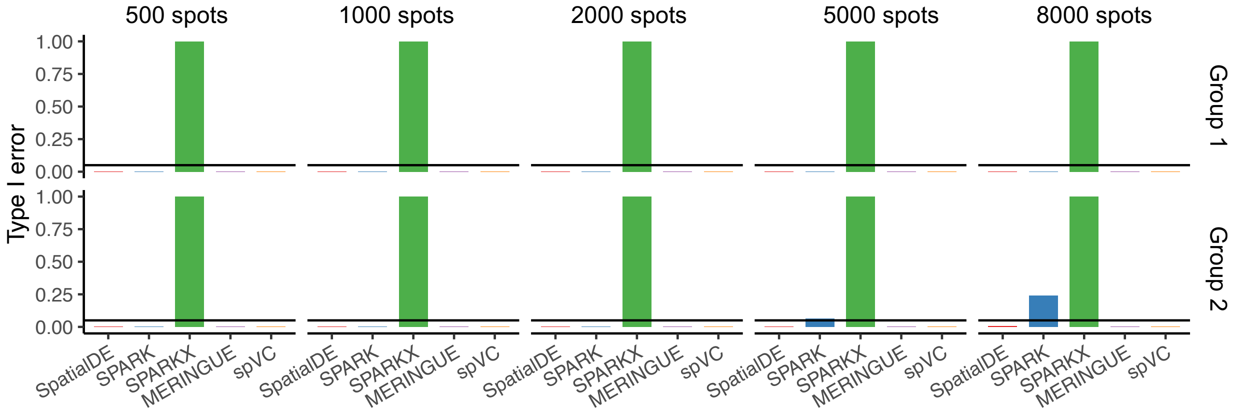

Fig. S1: Type I errors of the five methods on genes in Groups 1 and 2 for detecting residual spatial gene expression variation in the presence of covariates.

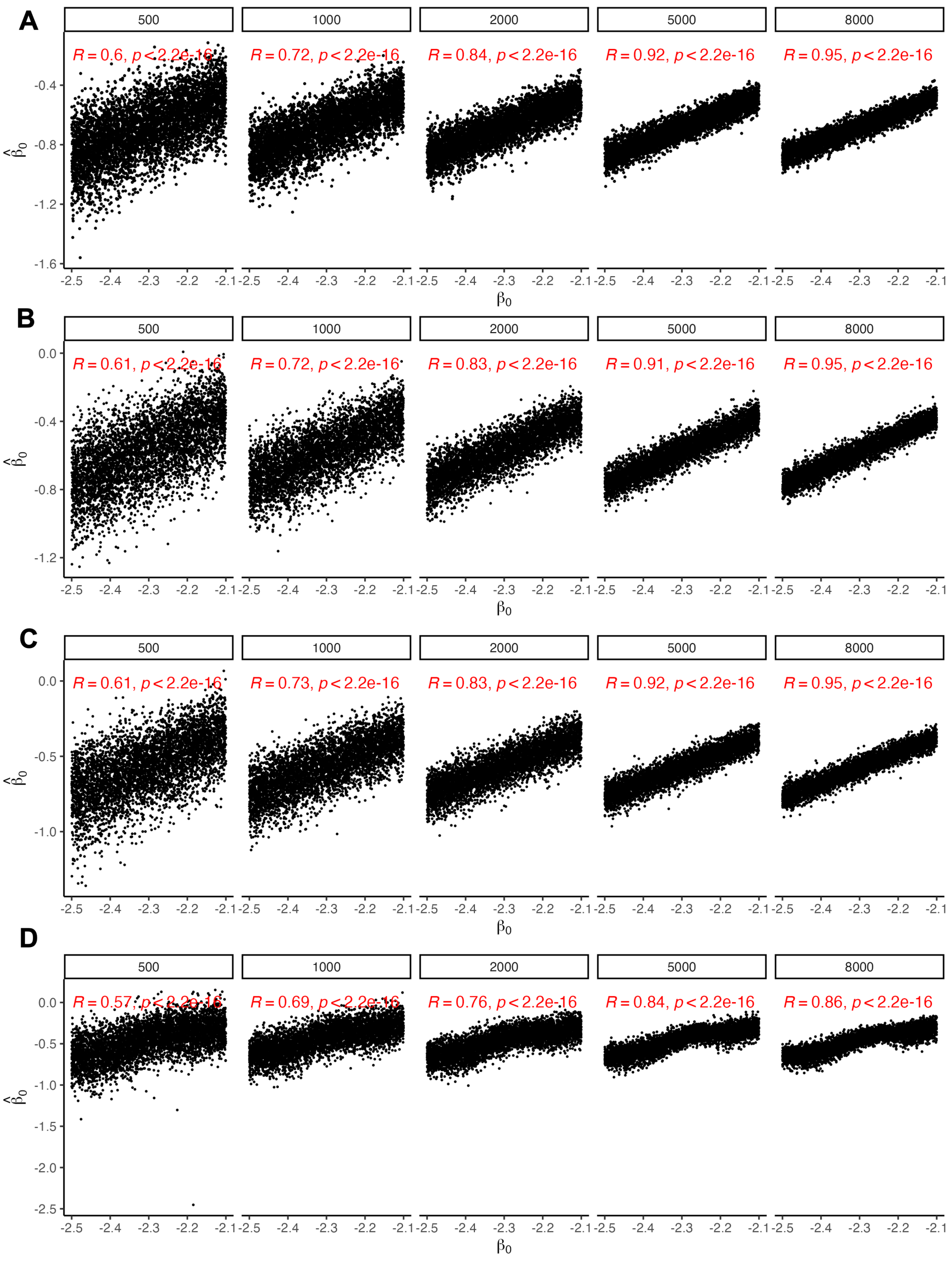

Fig. S2: Comparison between estimated intercept terms and true parameters used in simulation. (**A**): Group 1; (**B**): Group 2; (**C**): Group 3; (**D**): Group 4. Spot number is labelled on top of corresponding results. The Pearson correlation between true and estimated values and the corresponding P values are highlighted in red. It is worth noting that, since the data generation model and the spVC estimation model are not exactly the same, it is not appropriate to consider $\hat{\beta}_0$ from spVC as an estimator of β_0 used in simulation.

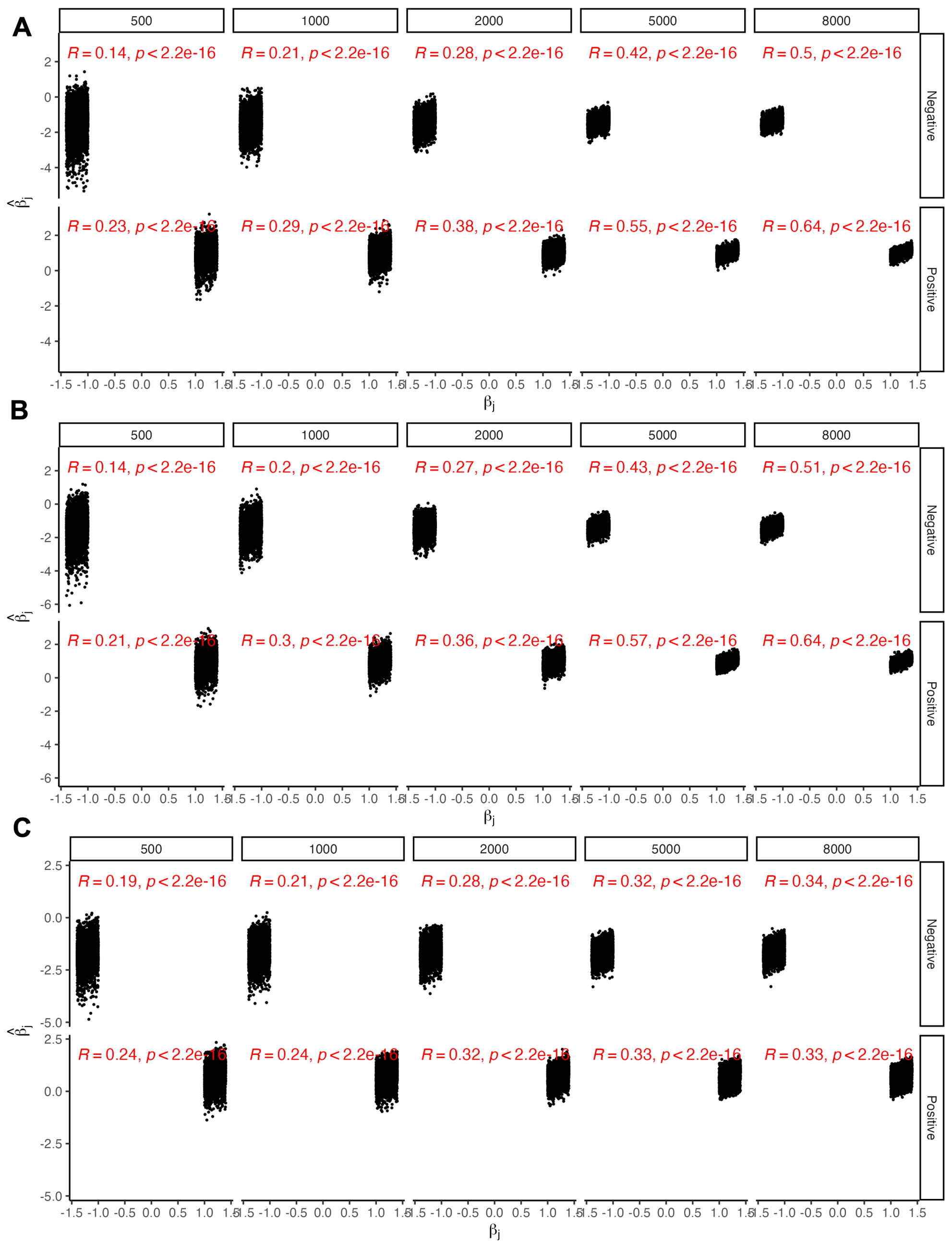

Fig. S3: Comparison between estimated covariates' constant effects and true parameters used in simulation. (**A**): Group 2; (**B**): Group 3; (**C**): Group 4. Results are separately displayed for positive and negative coefficients, and spot number is labelled on top of corresponding results. The Pearson correlation between true and estimated values and the corresponding P values are highlighted in red. It is worth noting that, since the data generation model and the spVC estimation model are not exactly the same, it is not appropriate to consider $\hat{\beta}_j$ from spVC as an estimator of β_j used in simulation.

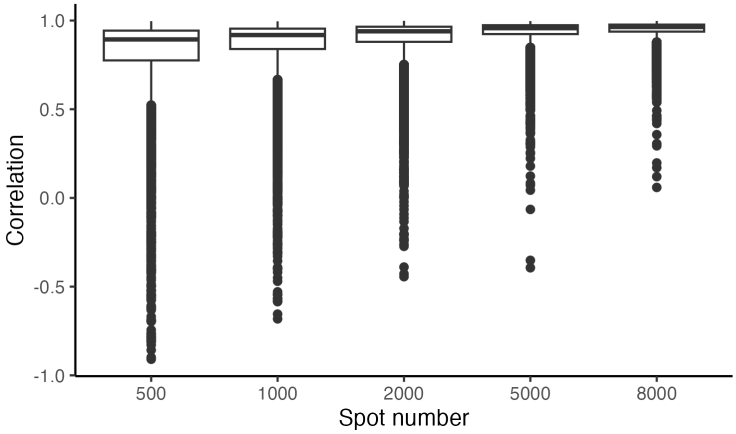

Fig. S4: Comparison between estimated and true spatial effects of covariates. For each gene, the true spatial effects function and the estimated spatial function were both evaluated at the observed spots, and the Pearson correlation between the two was calculated.

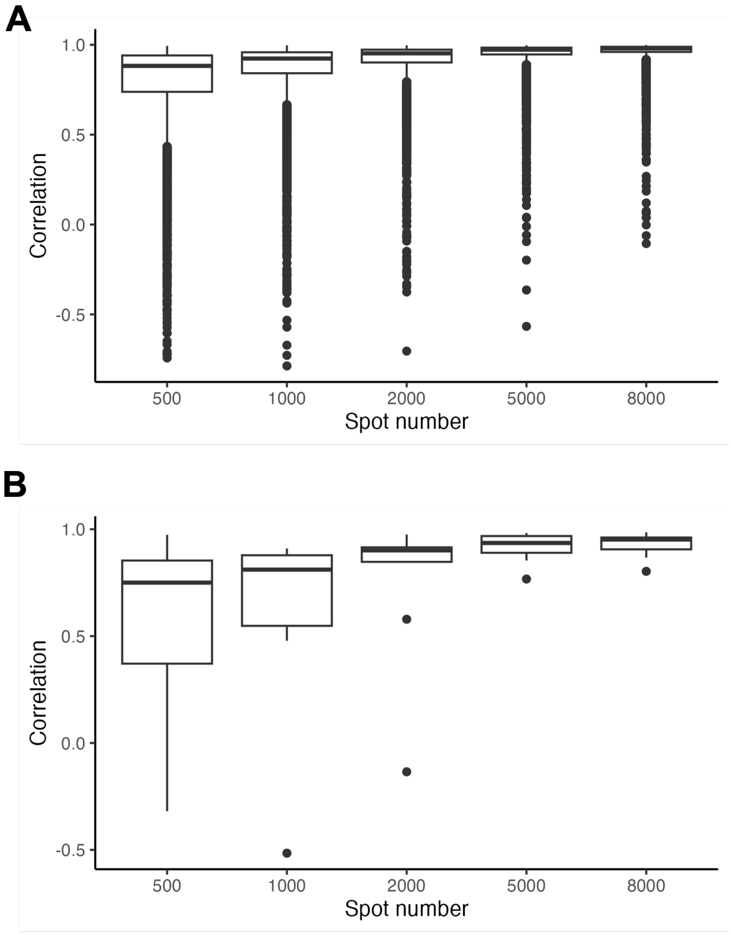

Fig. S5: Comparison between estimated and true residual spatial effects used in simulation. (**A**): Group 3; (**B**): Group 4. For each gene, the true residual spatial function and the estimated residual spatial function were both evaluated at the observed spots, and the Pearson correlation between the two was calculated.

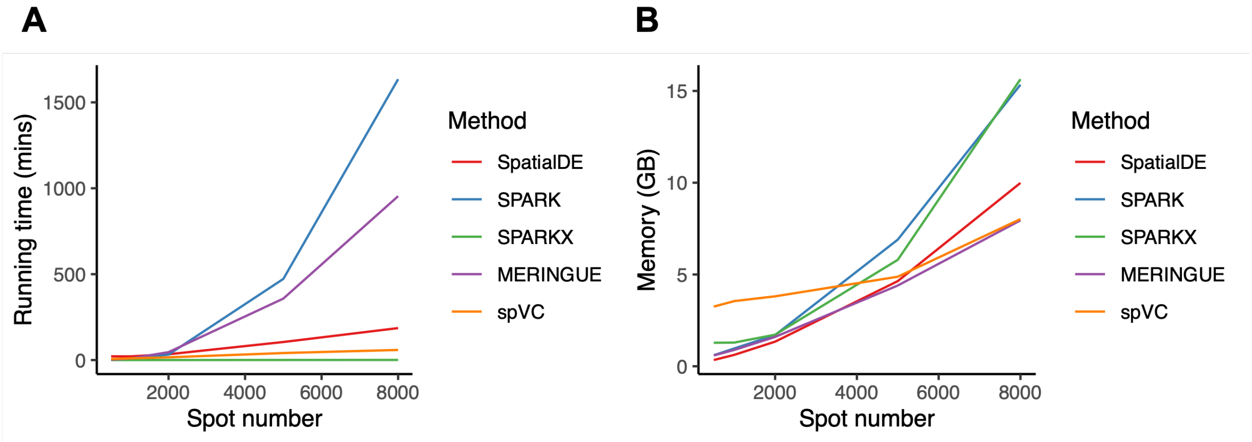

Fig. S6: Computational time (**A**) and maximum memory usage (**B**) on the simulated data. For methods that directly support parallel computation (SPARK, SPARK-X, and spVC), four cores were used.

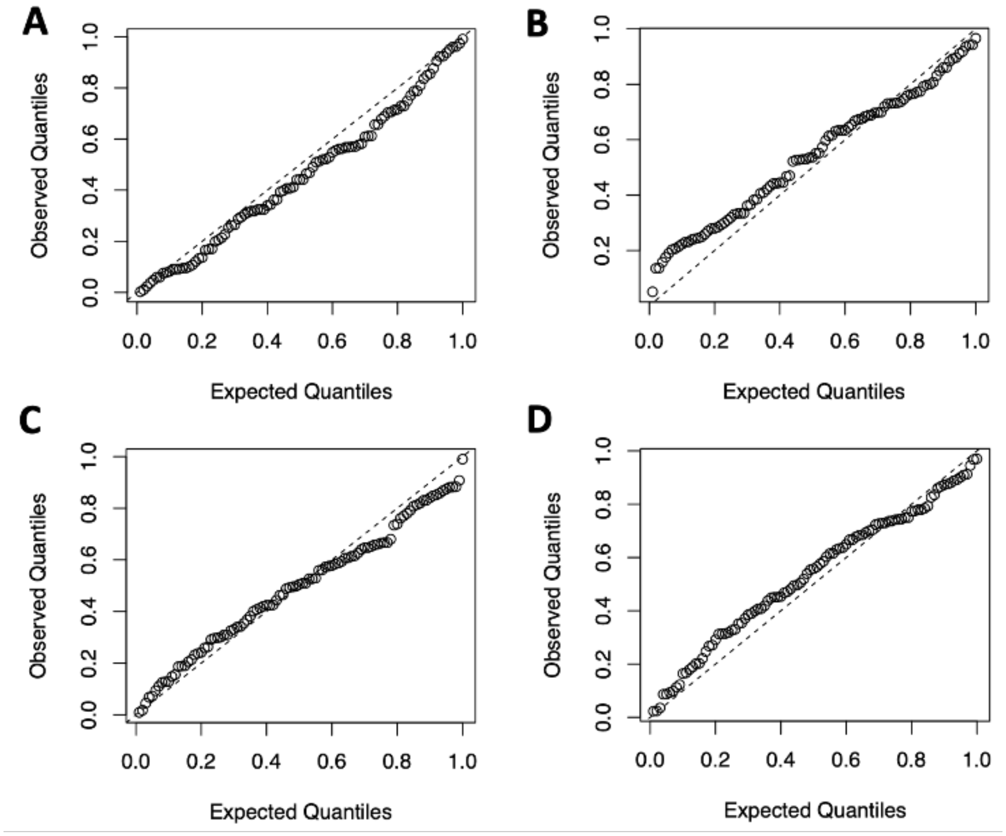

Fig. S7: Quantile-quantile plots of spVC's P values on null genes. **A-B**: Observed quantiles of P values vs. expected quantiles (based on the uniform distribution) for genes *GNB1* and *GPR88* in the human cortex data. **C-D**: Observed quantiles of P values vs. expected quantiles (based on the uniform distribution) for genes *Glrb* and *Kif1a* in the mouse cerebellum data.

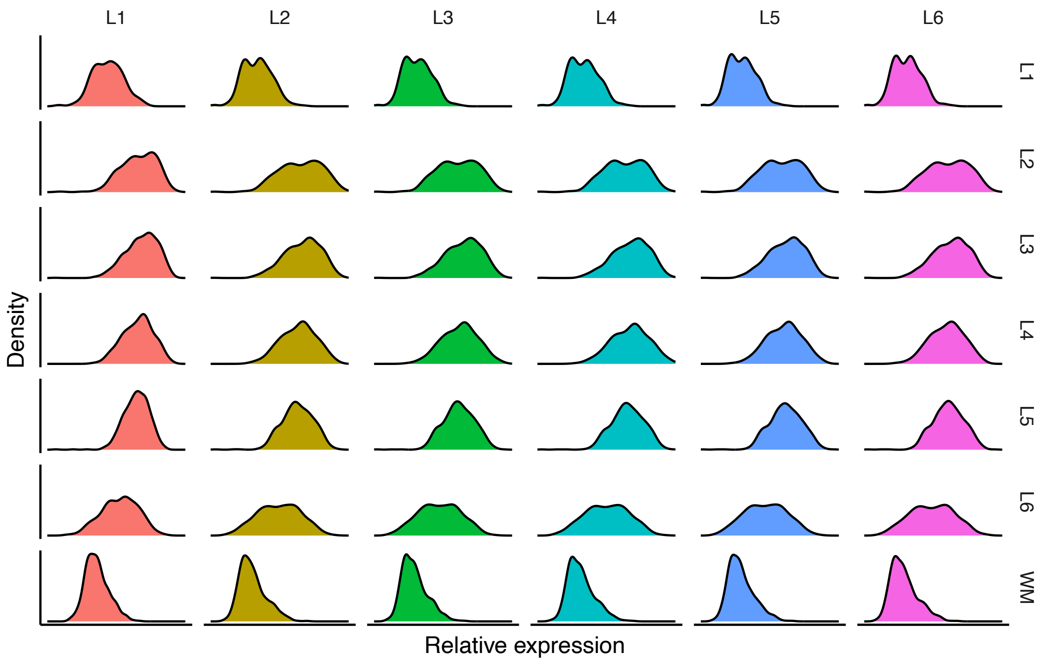

Fig. S8: Relative expression distribution of layer-associated genes (with positive coefficients) identified by spVC. The columns represent groups of genes associated with different layers. The rows denote which layers are used to plot the gene expression. For every gene group, the normalized expression levels of each gene were further scaled by the min-max normalization, and then for each spot, the average was taken across genes. For example, the first panel shows the distribution of relative expression of L1-associated genes in L1 spots.

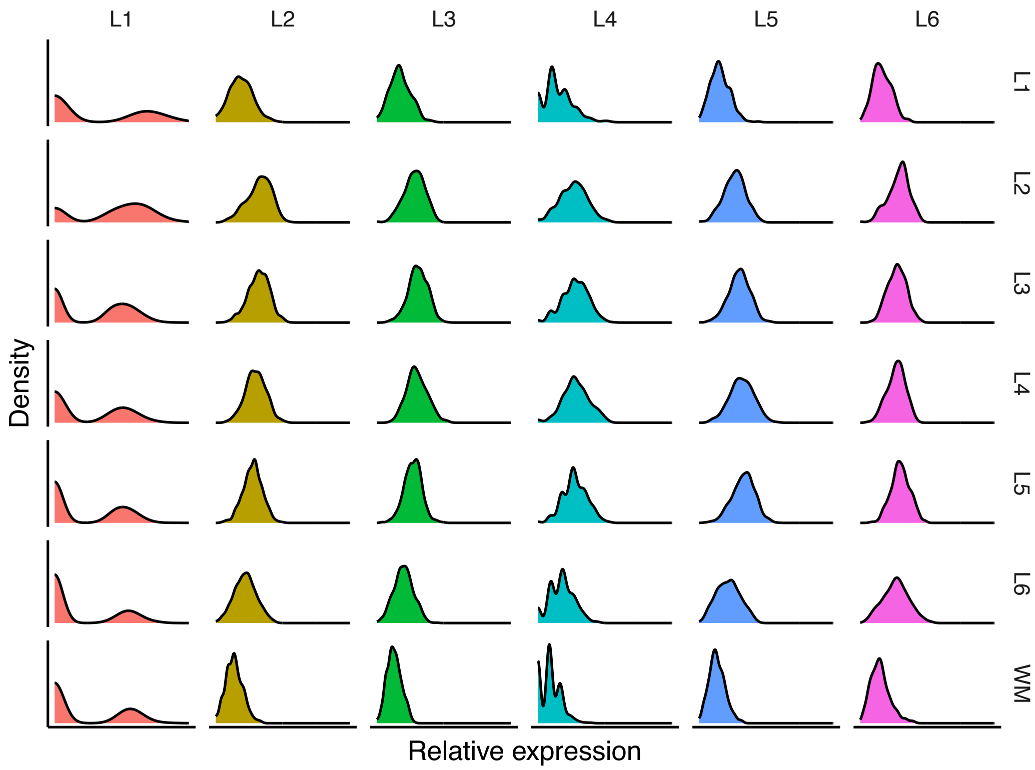

Fig. S9: Relative expression distribution of layer-associated genes commonly identified by previous literature and spVC. The columns represent groups of genes associated with different layers. The rows denote spots in which layers are used to plot the gene expression. For every gene group, the normalized expression levels of each gene were further scaled by the min-max normalization, and then for each spot, the average was taken across genes. For example, the first panel shows the distribution of relative expression of L1-associated genes in L1 spots.

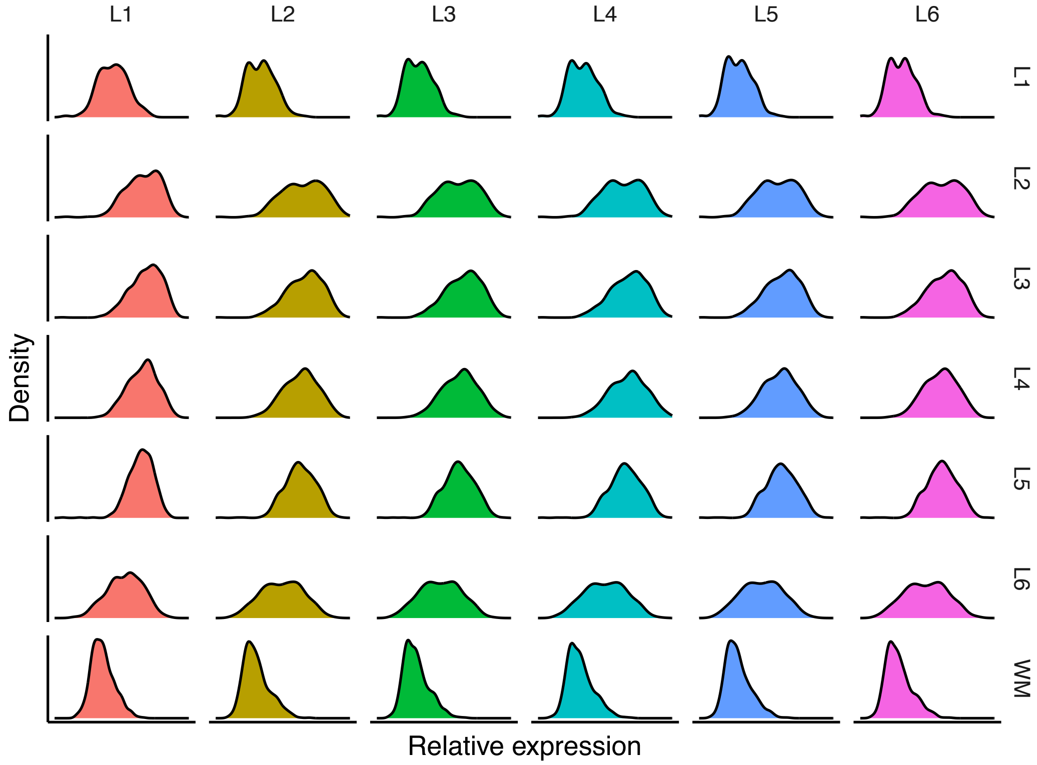

Fig. S10: Relative expression distribution of layer-associated genes identified by spVC but not included in the previously reported list. The columns represent groups of genes associated with different layers. The rows denote which layers are used to plot the gene expression. For every gene group, the normalized expression levels of each gene were further scaled by the min-max normalization, and then for each spot, the average was taken across genes. For example, the first panel shows the distribution of relative expression of L1-associated genes in L1 spots.

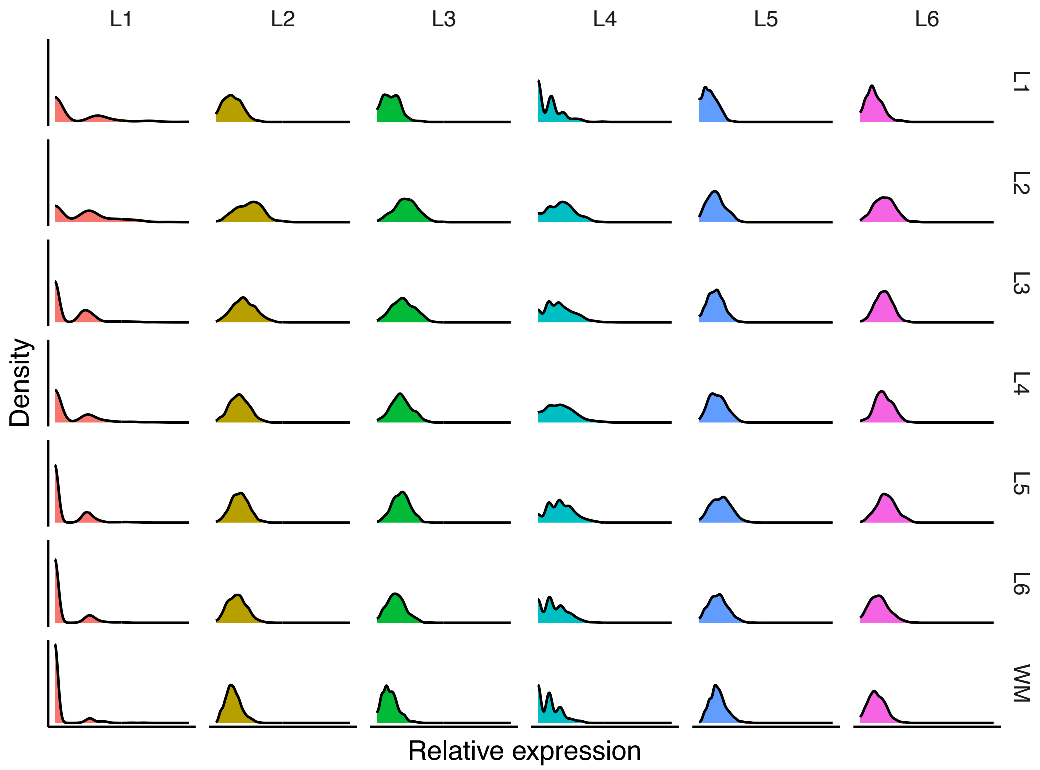

Fig. S11: Relative expression distribution of layer-associated genes included in the previously reported list but not reported by spVC. The columns represent groups of genes associated with different layers. The rows denote which layers are used to plot the gene expression. For every gene group and layer, the normalized expression levels of each gene were further scaled by the min-max normalization, and then for each spot, the average was taken across genes. For example, the first panel shows the distribution of relative expression of L1-associated genes in L1 spots.

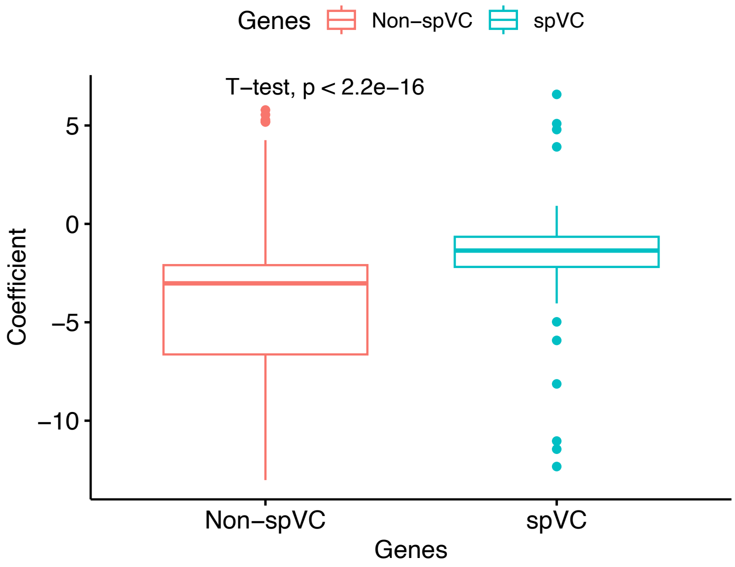

Fig. S12: Comparison between the 3022 genes which only had constant layer effects in spVC's results but were identified to have significant residual spatial effects by non-spVC methods with the 823 spVC genes. For each gene, the estimated residual spatial coefficients were evaluated on the observed spots and the average of their absolute values was calculated and used to make the boxplot (shown in the log scale). Genes identified by spVC had significant larger spatial effects than genes identified by non-spVC methods.

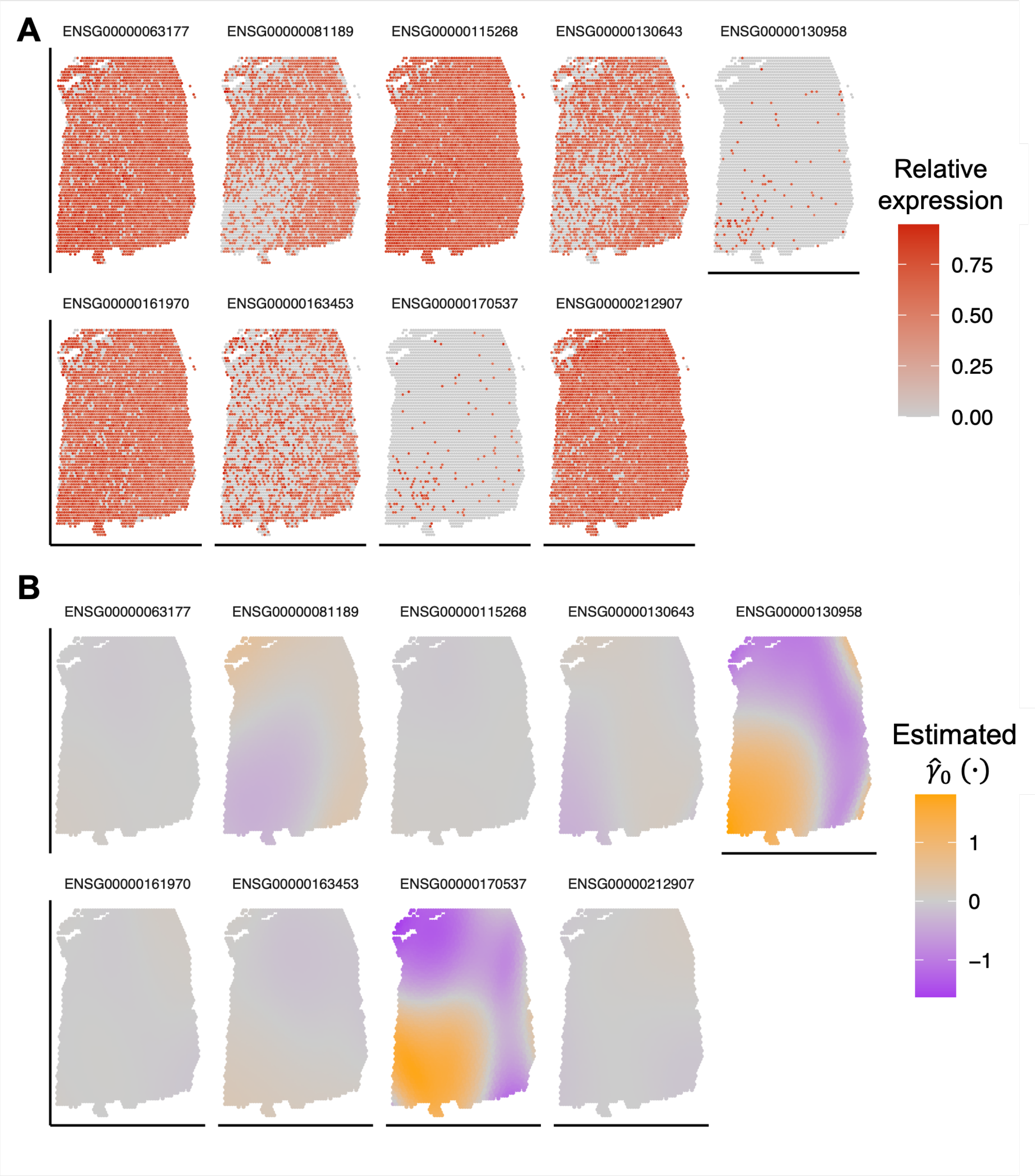

Fig. S13: Visualization of the nine genes identified to have significant residual spatial effects by all methods except for spVC. **A**: Observed relative expression of the nine genes. The read counts were normalized by library size, log-transformed, and then scaled by the min-max normalization to obtain the relative expression levels. **B**: Estimated residual spatial effects (by spVC) of the nine genes.

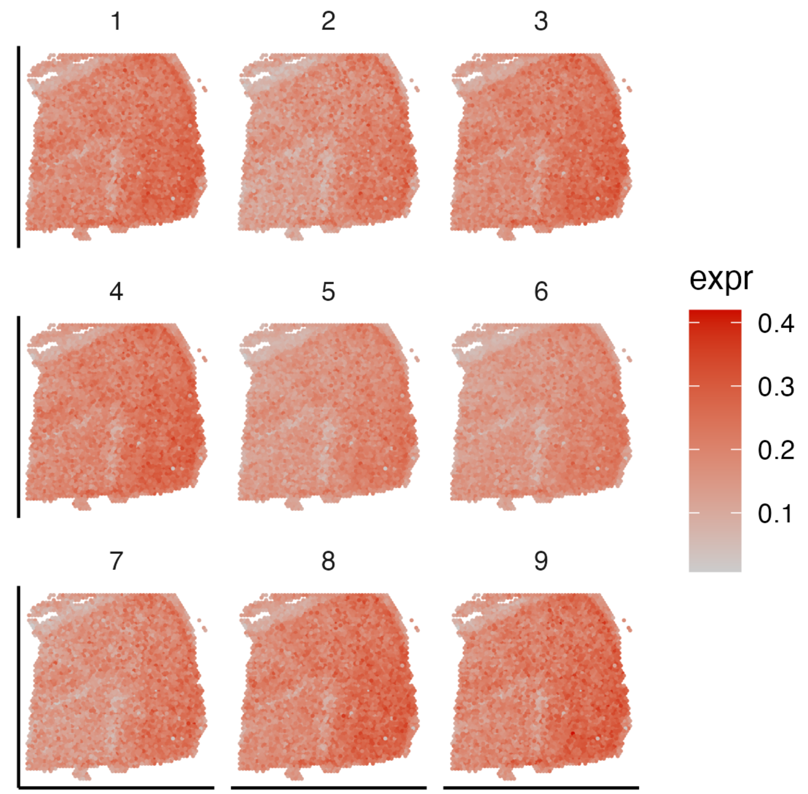

Fig. S14: Observed expression of spVC identified gene clusters. Displayed gene clusters are the same as shown in Figure 5. For every cluster, the normalized expression levels of each gene were further scaled by the min-max normalization, and then the average was taken across genes.

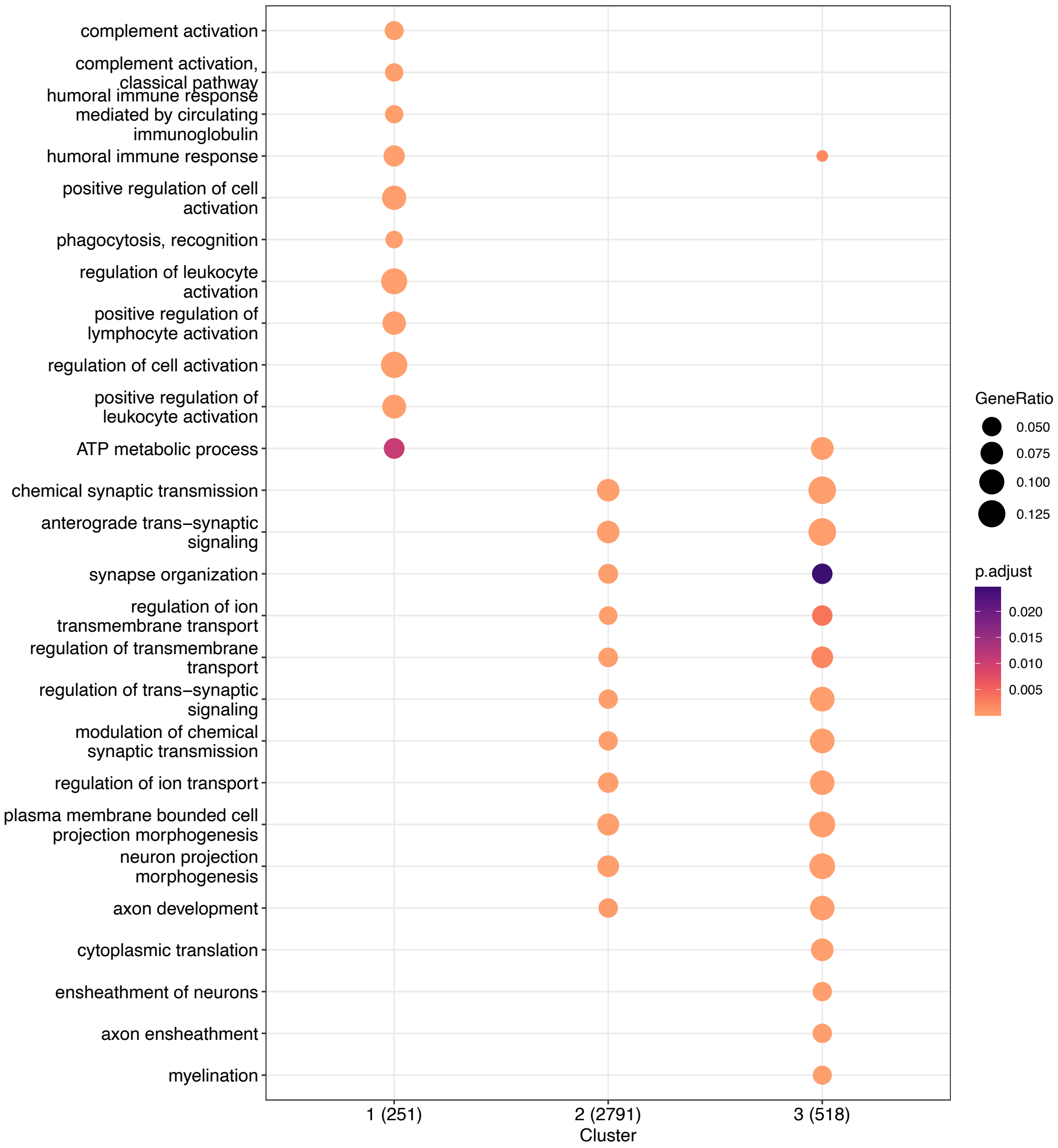

Fig. S15: GO enrichment analysis on the cortex data. Top 10 enriched GO terms in spatial-associated genes (cluster 1), layer-associated genes (cluster 2), and genes that were both spatial- and layer-associated (cluster 3) are shown in the figure. The numbers shown on the bottom were the numbers of genes in each cluster that were found in the GO database.

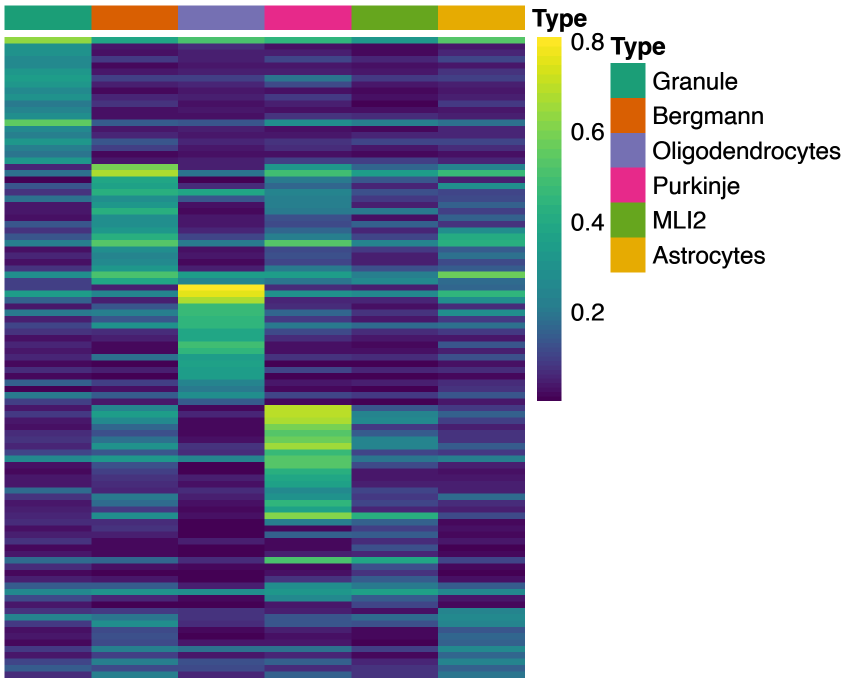

Fig. S16: Expression levels of top genes with significant covariate effects in the cerebellum data. For each cell type, the top 20 genes with the smallest P values were selected. For visualization, spots were assigned to cell types based on the largest cell type proportions. For each gene, its normalized gene expression level was further processed by the min-max normalization, and the resulting relative expression levels were averaged across spots within each cell type.

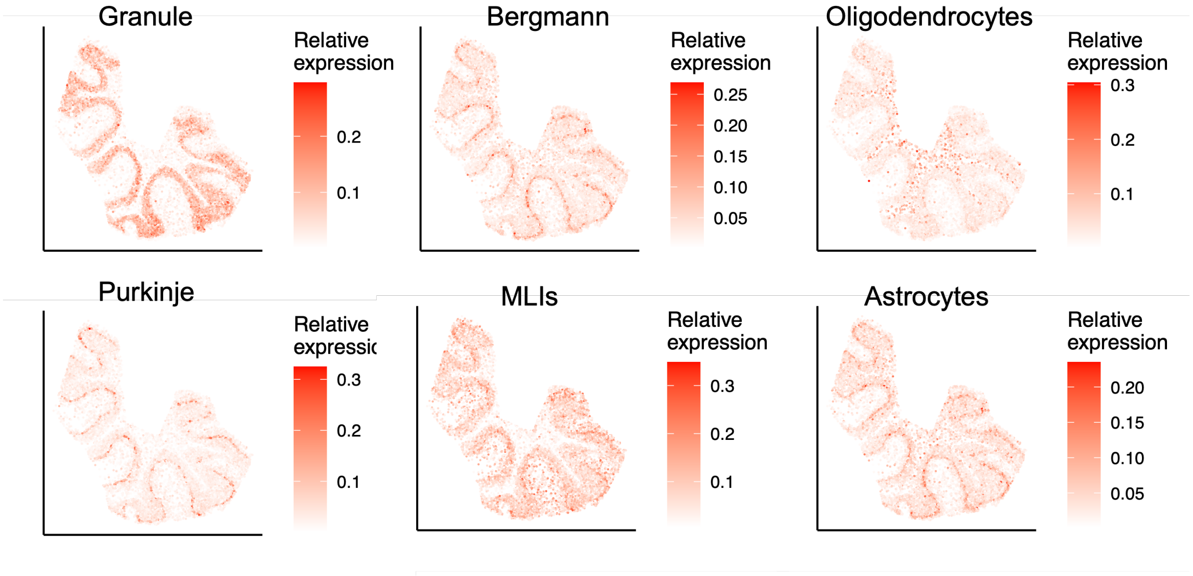

Fig. S17: Relative expression levels of cell-type-associated genes commonly identified by spVC and from the snRNA-seq data. For genes associated with each cell type, the normalized expression levels of each gene were further scaled by the min-max normalization, and then for each spot, the average was taken across genes.

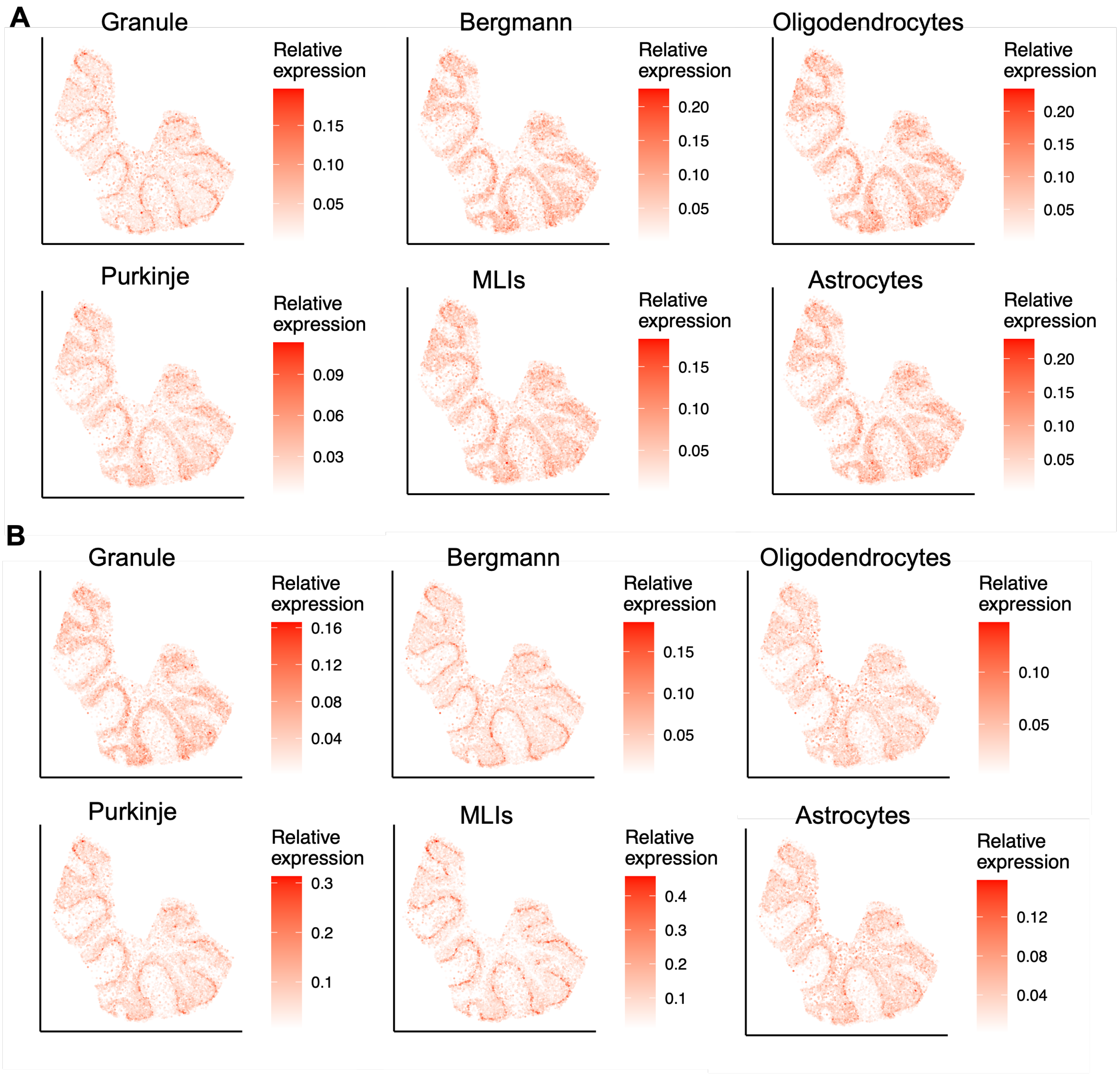

Fig. S18: Relative expression distribution of unique cell-type-associated genes. (**A**): Genes were uniquely reported from snRNA-seq data. (**B**): Genes were uniquely found by spVC. For genes associated with each cell type, the normalized expression levels of each gene were further scaled by the min-max normalization, and then for each spot, the average was taken across genes.

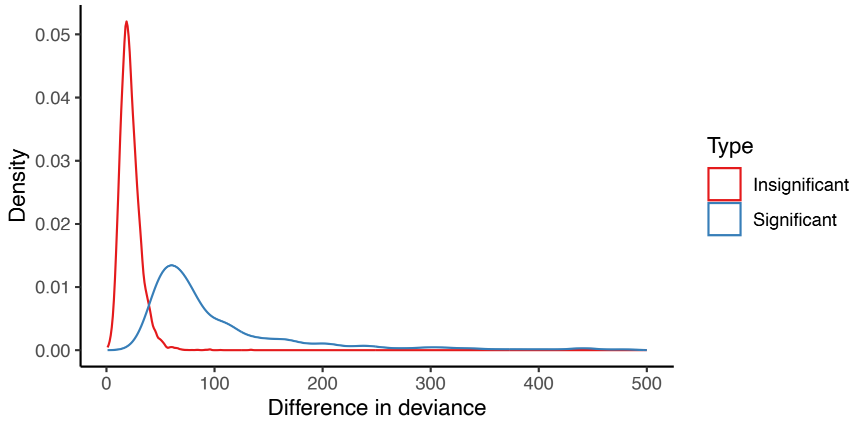

Fig. S19: Difference in deviance between spVC models without and with spatial effects. The red line shows the density for genes without significant residual spatial patterns after considering constant cell proportion effects. The blue line shows the density for genes with significant residual spatial patterns after considering constant cell proportion effects.

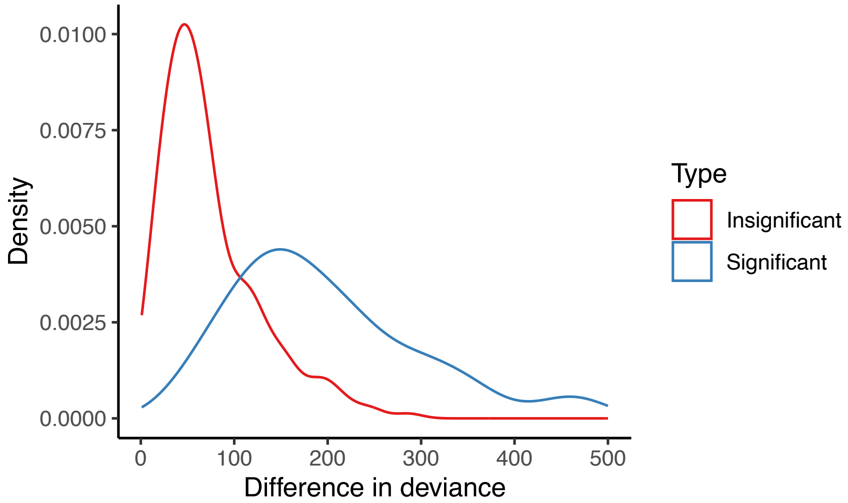

Fig. S20: Difference in deviance between spVC models without and with spatially varying effects of cell type proportions. The red line shows the density for genes without significant spatially varying effects of cell type proportions. The blue line shows the density for genes with significant spatially varying effects of cell type proportions.

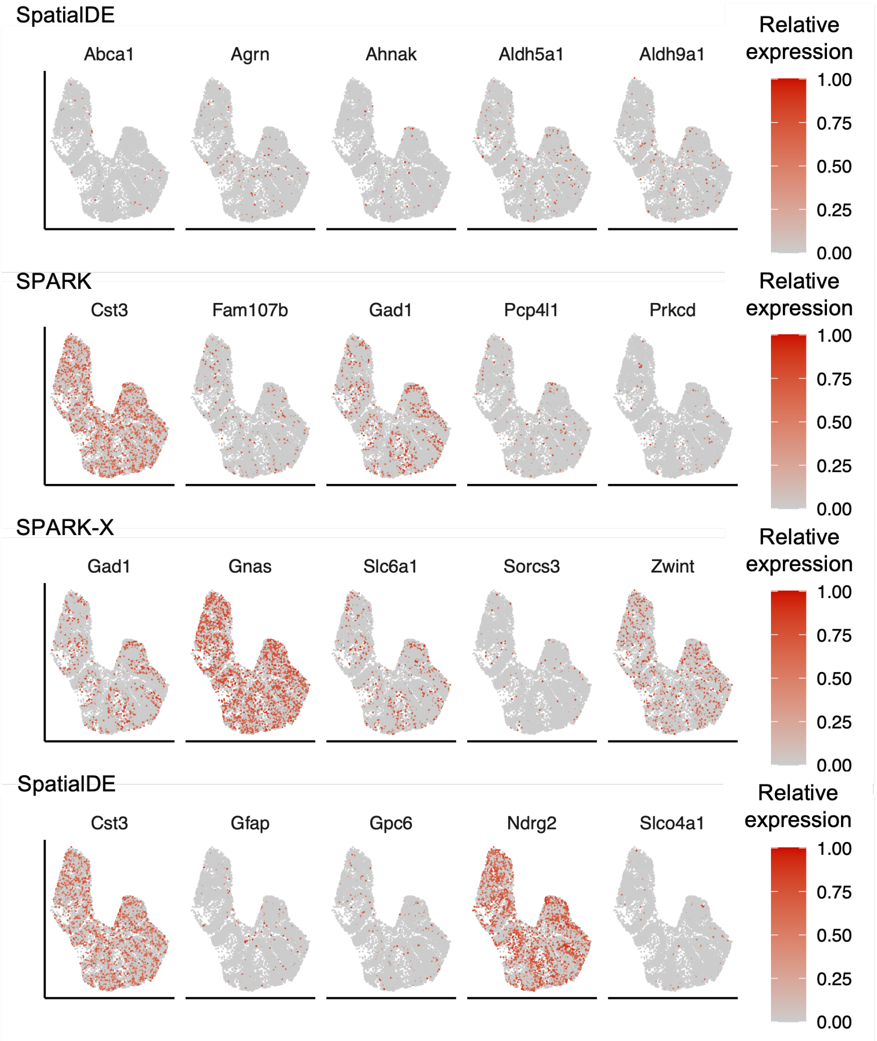

Fig. S21: Visualization of spatially variable genes identified by alternative methods. For each method, we obtained the genes that were reported to have significant residual spatial patterns but were only shown to be cell-type-associated by spVC, and visualized the relative

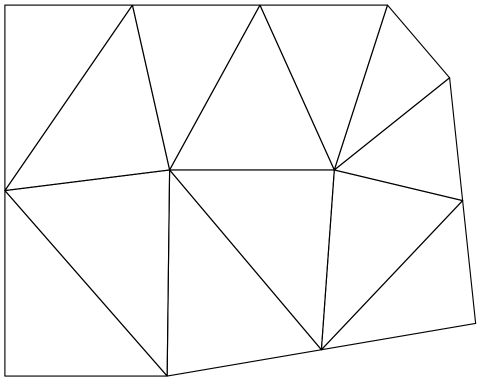

Fig. S22: Example triangulation of the human cortex dataset.

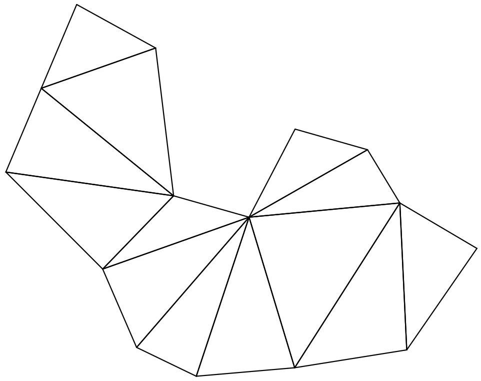

Fig. S23: Example triangulation of the mouse cerebellum dataset.

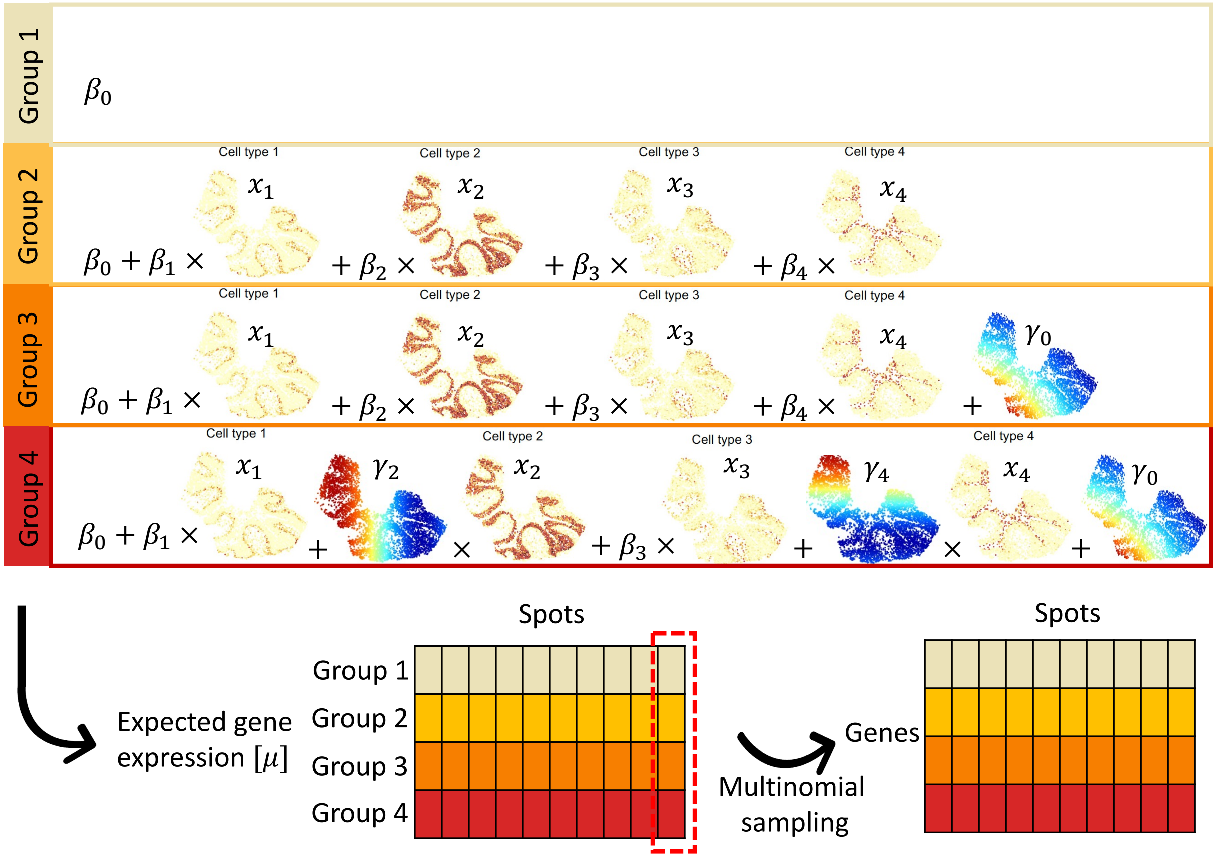

Fig. S24: Generation process of the simulated data. For a given spot number I, we first generated the two-dimensional spatial coordinates by randomly selecting I data points from the observed locations of the mouse cerebellum data. Next, we generated the expected expression levels of four groups of genes. Each group contained 5000 genes. We assumed that genes in Group 1 only depended on an intercept term; genes in Group 2 depended on an intercept and constant covariate effects; genes in Group 3 depended on an intercept, constant covariate effects, and residual spatial effects; genes in Group 4 depended on an intercept, constant covariate effects, spatially varying covariate effects, and residual spatial effects. After we obtained the expected expression levels of all genes across the I spots, the final read counts were simulated based on a multinomial sampling process for each spot.

Table S1: GO enrichment analysis on the cerebellum data. Top 20 GO terms that were only enriched in the cell-type-associated genes.

ID	Description	Adjuste *P* value
GO:0098609	cell-cell adhesion	1.93E-12
GO:0030029	actin filament-based process	9.97E-12
GO:0030036	actin cytoskeleton organization	3.46E-11
GO:0007015	actin filament organization	1.36E-08
GO:0042063	gliogenesis	5.36E-07
GO:1901699	cellular response to nitrogen compound	5.67E-07
GO:0031589	cell-substrate adhesion	6.37E-07
GO:0060284	regulation of cell development	9.67E-07
GO:1902903	regulation of supramolecular fiber organization	1.01E-06
GO:0071417	cellular response to organonitrogen compound	1.02E-06
GO:0043523	regulation of neuron apoptotic process	1.65E-06
GO:0007416	synapse assembly	2.06E-06
GO:0097435	supramolecular fiber organization	2.72E-06
GO:0051402	neuron apoptotic process	2.78E-06
GO:0030155	regulation of cell adhesion	2.93E-06
GO:0051241	negative regulation of multicellular organismal process	2.96E-06
GO:0006897	endocytosis	6.97E-06
GO:0007189	adenylate cyclase-activating G protein-coupled receptor signaling pathway	1.12E-05
GO:0043524	negative regulation of neuron apoptotic process	1.52E-05

Table S2: GO enrichment analysis on the cerebellum data. Top 20 GO terms that were only enriched in the spatial-associated genes.

ID	Description	Adjusted *P* value
GO:1904064	positive regulation of cation transmembrane transport	2.47E-10
GO:0034767	positive regulation of ion transmembrane transport	3.71E-10
GO:0006941	striated muscle contraction	3.26E-09
GO:0006936	muscle contraction	6.61E-09
GO:1903169	regulation of calcium ion transmembrane transport	9.39E-09
GO:1904427	positive regulation of calcium ion transmembrane transport	2.75E-08
GO:0051928	positive regulation of calcium ion transport	8.93E-08
GO:0060048	cardiac muscle contraction	1.19E-07
GO:0051209	release of sequestered calcium ion into cytosol	6.54E-07
GO:0051283	negative regulation of sequestering of calcium ion	8.36E-07
GO:0035725	sodium ion transmembrane transport	9.20E-07
GO:0051282	regulation of sequestering of calcium ion	1.06E-06
GO:0051208	sequestering of calcium ion	1.34E-06
GO:0060402	calcium ion transport into cytosol	1.46E-06
GO:0097553	calcium ion transmembrane import into cytosol	1.96E-06
GO:0032411	positive regulation of transporter activity	2.08E-06
GO:0051279	regulation of release of sequestered calcium ion into cytosol	3.05E-06
GO:0032414	positive regulation of ion transmembrane transporter activity	5.07E-06
GO:1901019	regulation of calcium ion transmembrane transporter activity	9.52E-06

Table S3: GO enrichment analysis on the cerebellum data. Top 20 GO terms that were only enriched in both cell-type-associated genes and spatial-associated genes.

ID	Description	Cell-type-associated	Spatial-associated
GO:0099536	synaptic signaling	8.80E-19	6.28E-13
GO:0099537	trans-synaptic signaling	8.80E-19	1.61E-12
GO:0007268	chemical synaptic transmission	1.04E-17	7.31E-13
GO:0098916	anterograde trans-synaptic signaling	1.04E-17	7.31E-13
GO:0034330	cell junction organization	2.74E-17	3.59E-05
GO:0043269	regulation of ion transport	1.85E-08	1.96E-15
GO:0098655	cation transmembrane transport	1.30E-06	4.22E-15
GO:0098662	inorganic cation transmembrane transport	1.43E-05	8.77E-15
GO:0098660	inorganic ion transmembrane transport	5.05E-07	2.63E-14
GO:0034220	ion transmembrane transport	1.36E-08	4.18E-14
GO:0006812	cation transport	7.80E-11	7.77E-14
GO:0034765	regulation of ion transmembrane transport	1.01E-04	7.77E-14
GO:0034762	regulation of transmembrane transport	2.90E-05	1.55E-13
GO:1904062	regulation of cation transmembrane transport	4.07E-03	3.81E-13
GO:0050808	synapse organization	8.96E-13	7.52E-05
GO:0000904	cell morphogenesis involved in differentiation	1.85E-12	1.52E-05
GO:0050877	nervous system process	4.81E-11	3.11E-12
GO:0030001	metal ion transport	8.74E-08	5.01E-12
GO:0006873	cellular ion homeostasis	2.54E-02	5.36E-12

Table S4: Comparison of significant spatial genes (after accounting for cell type proportions) identified by different methods. For the comparison, the spVC genes were divided into three groups: 4459 genes that were only cell-type-associated but did not have significant residual spatial patterns, 4 genes that had significant residual spatial patterns but were not cell-type-associated, and 300 genes that were cell-type-associated and had significant residual spatial patterns. The number of genes shared by the other methods and the spVC genes were summarized.

	spVC Cell-type-associated (4459 genes)	spVC Spatial-associated (4 genes)	spVC Both (300 genes)
SPARK Spatial-associated (820 genes)	480	1	257
SPARKX Spatial-associated (3818 genes)	2407	3	263
SpatialDE Spatial-associated (2183 genes)	972	1	186
MERINGUE Spatial-associated (278 genes)	130	0	91
