# Supplementary material for: spVC for the detection and interpretation of spatial gene expression variation
Source: Genome Biol. 2024 Apr 19;25:103. doi: 10.1186/s13059-024-03245-3 (PMC11027374; doi:10.1186/s13059-024-03245-3)
Supplement: Supplementary file 2 — Additional file 2. Supplementary methods. [file 13059_2024_3245_MOESM2_ESM.pdf]

Supplementary Methods

Details of the bivariate spline over triangulation (BPST) method

In this paper, we consider the spatial domain Ω as a polygon of arbitrary shape, which can be partitioned into finitely many triangles. According to [42], a collection $\triangle = \{T_1, ..., T_K\}$ of K triangles is a triangulation of Ω provided that any nonempty intersection between a pair of triangles in $\triangle$ is either a shared vertex or a shared edge. See Supplementary Figures S22 and S23 for the triangulations of the human cortex dataset and mouse cerebellum dataset.

For a triangle with non-zero area $T \in \triangle$ and any fixed point $\boldsymbol{s} \in \mathbb{R}^2$, let b_1, b_2, and b_3 be the barycentric coordinates of $\boldsymbol{s}$ relative to T. The Bernstein basis polynomials of degree $d \geq 1$ relative to triangle T is defined as $B_{ijk}^{T,d}(\boldsymbol{s}) = \frac{d}{i!j!k!} b_1^i b_2^j b_3^k$, $i+j+k = d$. For any integer $d \geq 1$ and triangle T, let $\mathbb{P}_d(T)$ be the space of all polynomials of degree less than or equal to d on T. See Supplementary Figure S25 for an example of Bernstein basis polynomials with degree $d = 2$ on a triangle T. Then, any polynomial $\zeta \in \mathbb{P}_d(T)$ can be written as $\zeta|_T = \sum_{i+j+k=d} \gamma_{ijk}^T B_{ijk}^{T,d}$, where the coefficients $\boldsymbol{\gamma}_{\tau} = \{\gamma_{ijk}^T, i+j+k = d\}$ are called B-coefficients of ζ.

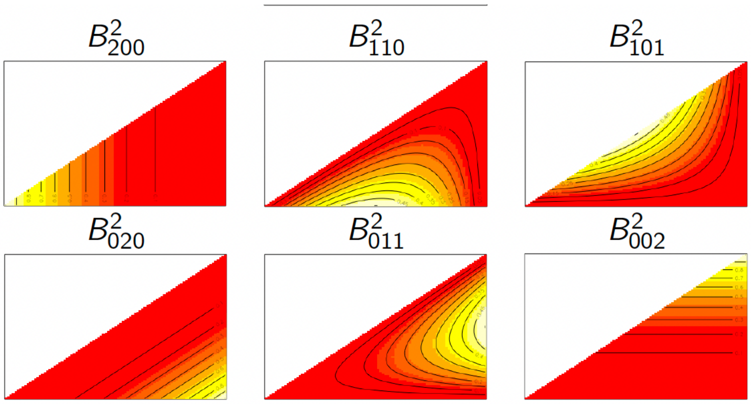

Fig. S25: Bernstein basis polynomials of degree $d = 2$ for T.

For any integer $r \geq 0$, let $\mathbb{C}^r(\Omega)$ be the collection of all r-th continuously differentiable functions over Ω. Given a triangulation $\triangle$, we define the spline space of degree d and smoothness r over $\triangle$ as $\mathbb{S}_d^r(\triangle) = \{\zeta \in \mathbb{C}^r(\Omega) : \zeta|_T \in \mathbb{P}_d(T), T \in \triangle\}$. Let $\{B_m\}_{m \in \mathcal{M}}$ be the set of bivariate Bernstein basis polynomials for $\mathbb{S}_d^r(\triangle)$, where $\mathcal{M}$ is an index set of $|\mathcal{M}| = K(d+1)(d+2)/2$ basis functions. We centralize the bivariate Bernstein basis polynomials by $B_m(\boldsymbol{s}) = B_m(\boldsymbol{s}) - \int B_m(\boldsymbol{s}) d\boldsymbol{s}$. Then we can represent any function $\zeta \in \mathbb{S}_d^r(\triangle)$ using the following basis expansion:

$$\zeta(\mathbf{s}) = \sum_{m \in \mathcal{M}} B_m(\mathbf{s}) \gamma_m = \mathbf{B}(\mathbf{s})^\top \boldsymbol{\gamma},$$

where $\boldsymbol{\gamma}^\top = (\gamma_m, m \in \mathcal{M})$ is the spline coefficient vector. For smooth join between two polynomials on adjoining triangles, we need to impose some linear constraints on the spline coefficients $\boldsymbol{\gamma}$. To be more specific, we assume that $\mathbf{H}$ satisfies $\mathbf{H}\boldsymbol{\gamma} = \mathbf{0}$, where $\mathbf{H}$ is the matrix that collects the smoothness conditions across all the shared edges of triangles. An example of $\mathbf{H}$ is given in Section B.2.1 in the supplementary materials of [42]. The above bivariate spline

basis functions can be constructed via the R package BPST [40].
